# A practical nomogram and risk stratification system predicting the cancer‐specific survival for patients with early hepatocellular carcinoma

**DOI:** 10.1002/cam4.3613

**Published:** 2020-12-06

**Authors:** Bing Yan, Bing‐Bing Su, Dou‐Sheng Bai, Jian‐Jun Qian, Chi Zhang, Sheng‐Jie Jin, Guo‐Qing Jiang

**Affiliations:** ^1^ Department of Hepatobiliary Surgery Clinical Medical College Yangzhou University Yangzhou China; ^2^ Department of Hepatobiliary Surgery The Second Clinical College Dalian Medical University Dalian China

**Keywords:** cancer‐specific survival, hepatocellular carcinoma, nomogram, predict, risk stratification

## Abstract

**Background:**

Our purpose was to establish and validate a nomogram model in early hepatocellular carcinoma (HCC) patients for predicting the cancer‐specific survival (CSS).

**Methods:**

We extracted eligible data of relevant patients between 2010 and 2015 from the Surveillance, Epidemiology, and End Results (SEER) database. Further, we divided all patients into two groups (training and validation cohorts) at random (7:3). Nomogram was established using effective risk factors based on univariate and multivariate analysis. The effective performance of nomogram was evaluated using concordance index (C‐index), calibration plots, decision curve analysis (DCA), and receiver operating characteristic curve (ROC).

**Results:**

We selected 3620 patients with early HCC including the training cohort (70%, 2536) and the validation cohort (30%, 1084). The nomogram‐related C‐indexes were 0.755 (95% CI: 0.739–0.771) and 0.737 (95% CI: 0.712–0.762), in the training and validation cohorts, respectively. The calibration plots showed good consistency of 3‐and 5‐year CSS between the actual observation and the nomogram prediction. The 3‐, 5‐year DCA curves also indicated that the nomogram has excellent clinical utility. The 3‐, 5‐year area under curve (AUC) of ROC in the training cohort were 0.783, 0.779, respectively, and 0.767, 0.766 in the validation cohort, respectively. With the establishment of nomogram, a risk stratification system was also established that could divide all patients into three risk groups, and the CSS in different groups (i.e., low risk, intermediate risk, and high risk) had a good regional division.

**Conclusions:**

We developed a practical nomogram in early HCC patients for predicting the CSS, and a risk stratification system follow arisen, which provided an applicable tool for clinical management.

## Graphical abstract

Our purpose was to establish and validate a nomogram model in early hepatocellular carcinoma (HCC) patients for predicting the cancer‐specific survival (CSS). We developed a practical nomogram in early HCC patients for predicting the CSS, and a risk stratification system follow arisen, which provided an applicable tool for clinical management.

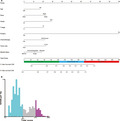

## 1 INTRODUCTION

In primary liver cancer, hepatocellular carcinoma (HCC) accounts for 75%–85%. HCC rank as the sixth most common cancer and the fourth leading reason for cancer‐related mortality worldwide.^1^ Although HCC was more common cancer in the developing countries, since the early 1980s, the incidence rate of HCC has almost tripled in the United States, and it has the most important contributor to cause of cancer‑related mortality rising.^2^, ^3^ Most HCC patients are discovered at an intermediate‐to‐advanced stage and has a poor prognosis.^4^ Nevertheless, with the improvement of diagnostic techniques and the enforcement of routine screening for high‐risk patients, a greater proportion of early HCC patients are diagnosed.^5^ Hence, the survival prognosis of patients with early HCC remains a significant concern.

As we all know, the American Joint Committee on Cancer (AJCC) TNM staging system is often used to evaluate the survival prognosis of HCC patients.^6^ Even so, there is the study show that the TNM staging system was not effective in predicting the survival prognosis of HCC.^7^ So far, many risk factors for affecting the survival prognosis for patients with HCC have been discovered, including age,^8^ race,^9^ marital status,^10^ histological grade,^11^ chemotherapy,^12^ surgery,^13^ tumor size,^14^ and so on. Of note, compared with the TNM staging system, these aforementioned factors may be more credible in the prediction of survival, and readily available. Therefore, we expect to establish a novel model which could combine all the effective prognostic factors, to provide a more accurate prediction for survival of patients with early HCC.

Nomogram‐based clinical modeling is a reliable statistical predictive model, through comprehensive analysis of all tumor‐related risk factors, it can accurately calculate and predict the survival rate of different patients.^15^, ^16^ More importantly, nomogram rely on intuitive advantages and numerical strength, that facilitate the probability analysis of tumor‐related risk factors. To the best of our knowledge, numerous nomogram prediction models for HCC have been structured.^17^, ^18^, ^19^ However, little study has been done, using the SEER database, to construct the nomogram model to predict CSS for early HCC. In fact, a prognostic nomogram for CSS of early HCC patients is to be adequately developed and validated. Aim at patients with early HCC, we deem that a more practical, reliable and specific prediction model for predicting CSS is necessary.

Our study aimed to develop and validate a practical nomogram for predicting the CSS of patients with early HCC by integrating some significant variables in order that clinicians can make better decisions.

## 2 MATERIALS AND METHODS

### 2.1 Patients selection and study variables

Data of clinical‐related for patients diagnosed with HCC between 2010 and 2015 were extracted from the SEER 18 registry database (1973–2015) by SEER*Stat 8.3.6 software. Data included baseline demographic, tumor characteristics, therapeutic method, stage at diagnosis, survival time, and so on. Inclusion criteria included: patients with early HCC (8170, 8171, 8172, 8173, 8174, and 8175; tumor size ≤5 cm; T1/T2, N0, and M0). Exclusion criteria included: (a) unknown whether surgery was performed; (b) no first tumor; (c) unknown marital status or domestic partner; (d) unknown race; (e) unknown histological grade; (f) unknown insurance; (g) other tumors death and unknown cause of death. Ultimately, 3620 patients with early HCC from the SEER database were included and further analyzed in our study. Below variables were included analysis: baseline demographics (race, sex, age at diagnosis, marital status, insurance, income, survival months, and vital status), tumor features (pathological grade, TNM stage, and tumor size), and treatment strategy (surgery, radiotherapy, and chemotherapy). The patients in the study included three race groups: white, black, or other (American Indian/Alaskan Native, Asian/Pacific Islander); age of patients were grouped as two groups: ≤65 and >65 years old; in treatment, included with or without surgery, with or without radiotherapy, with or without and unknown chemotherapy; and marital status were classified into “married,” “single (never married),” “divorced/separated,” and “widowed.” In addition, we analyzed using the seventh edition AJCC TNM staging.

### 2.2 Construction and validation of the nomogram model

We randomly divided the total cases into two groups in a 7:3 ratio, which consisted of the training cohort (*n* = 2536, 70% of total cases) and validation cohort (*n* = 1084, 30% of total cases). The nomogram model was structured using the training cohort, and the validation cohort was used for validation. We performed univariate and multivariate analysis to obtain for significant factors that significantly affect CSS (*p* < 0.05) and further construction of nomogram. We used the concordance index (C‐index), calibration curves, decision curve analyses (DCA), and receiver operating characteristics curve (ROC) for the validation of the nomogram. The C‐indexes were used to reflect the performance and predictive accuracy of the nomogram. Calibration plots at 3 and 5 years (1000 bootstrap resamples) were drawn to compare the predicted CSS with the CSS observed in our study, and the 45‐degree line is used as the actual result of the first‐rank model. ROCs were generated for the sensitivity and specificity of nomogram. The values of the C‐index or AUC ranges from 0.5 to 1.0, with 0.5 representing the random probability and 1.0 representing the perfect ability to accurately judge the result. DCAs were drawn to evaluate the clinical practicability of the nomogram. Moreover, according to the cutoff value that the total score calculated from the nomogram among the training cohort patients, all eligible patients were classified into three groups (i.e., low risk, intermediate risk, and high risk). Kaplan–Meier curves and the log‐rank test were used to compare the CSS of patients in the different groups. Data were extracted using SEER*Stat software version 8.3.6. We used statistical software IBM SPSS Statistics, Version 25.0 (SPSS, Inc) to execute univariate and multivariate regression analyses. The C‐indexes, calibration plots, ROCs, and DCA curves were generated using R version 3.6.3 with relevant packages. The cutoff value that the total score was calculated by X‐Tile, version 3.6.1. The Forest plot of showing univariate and multivariate analysis results and Kaplan–Meier curves were drawn using GraphPad Prism 8. When *p*‐value is less than 0.05, it is statistically significant.

## 3 RESULTS

### 3.1 Patient characteristics

**FIGURE 1:**
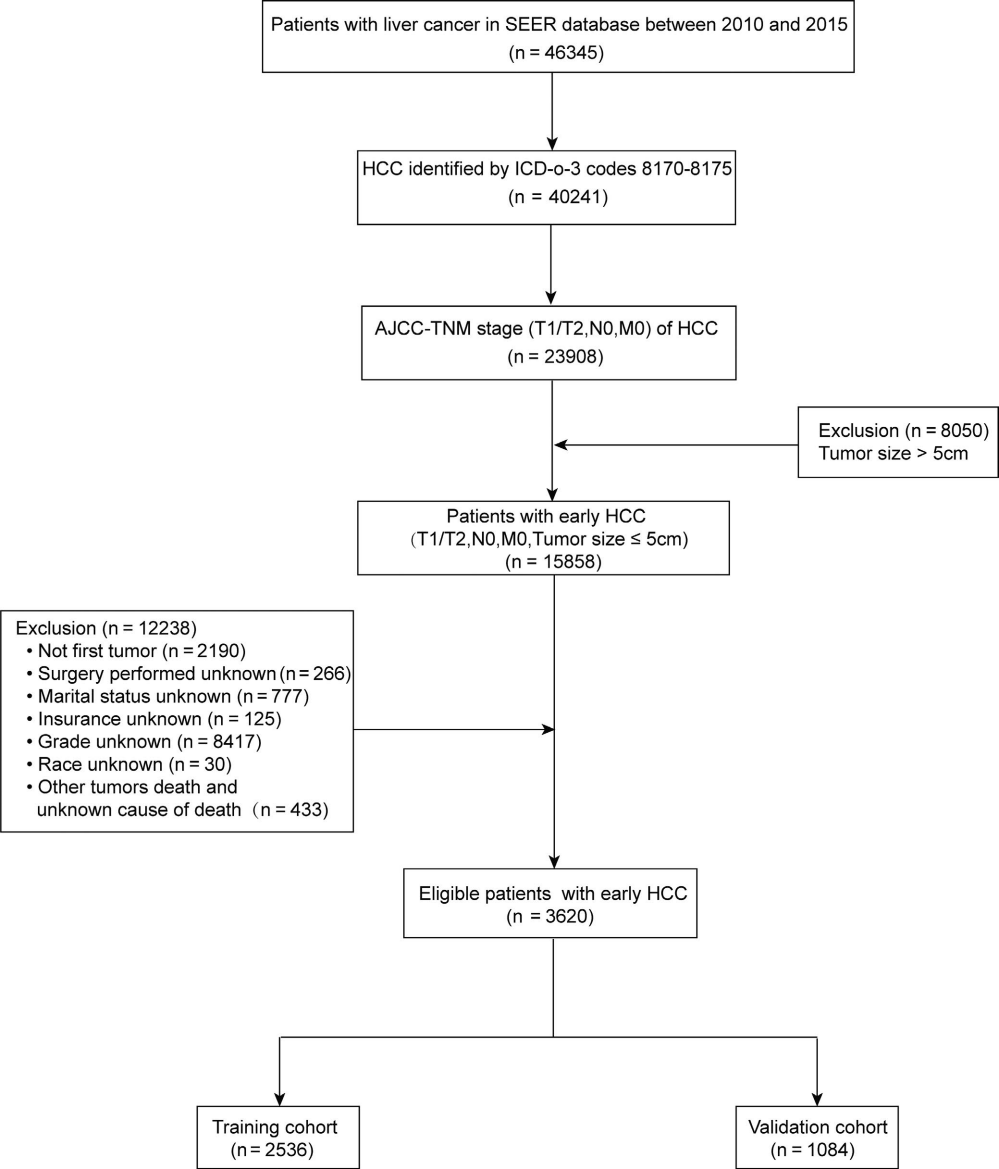
Flow diagram of the early hepatocellular carcinoma patients with training and validation cohorts

A total of 3620 eligible patients with HCC were included in our study, including the training cohort (*n* = 2356), validation cohort (*n* = 1084) (Figure 1). Of these patients, 2711 (74.9%) were male, 2436 (67.3%) were White, 2118 (58.5%) were Married, 2761 (76.3%) were Insured, 3083 (85.2%) were grade I/II, and 2227 (61.5%) were T1 stage. Almost half 1777 (49.1%) of the patients had tumors smaller than 3 cm. Most patients 2537 (70.1%) were performed surgery, while 51 (1.4%) received radiotherapy and 1148 (31.7%) received Chemotherapy. The baseline demographics and characteristics of patients with early HCC in the training cohort and internal validation cohort are summarized in Table 1, and there was no significant difference in the two groups.

**TABLE 1:** Baseline demographic and clinical characteristics of early HCC patients in the training cohort and validation cohort

Characteristic	All cohort	Training cohort	Validation cohort	*P*‐value ^a^
	*n* = 3620	*n* = 2536	*n* = 1084	
	N(%)	N(%)	N(%)	
Age				0.051
≤65	2413 (66.7%)	1662 (65.5%)	751 (69.3%)	
>65	1207 (33.3%)	874 (34.5%)	333 (30.7%)	
Gender				0.307
Female	909 (25.1%)	649 (25.6%)	260 (24.0%)	
Male	2711 (74.9%)	1887 (74.4%)	824 (76.0%)	
Race				0.278
White	2436 (67.3%)	1692 (66.7%)	744 (68.6%)	
Black	464 (12.8%)	322 (12.7%)	142 (13.1%)	
Other	720 (19.9%)	522 (20.6%)	198 (18.3%)	
Grade				0.744
I/II	3083 (85.2%)	2163 (85.3%)	920 (84.9%)	
III/IV	537 (14.8%)	373 (14.7%)	164 (15.1%)	
T stage				0.462
T1	2227 (61.5%)	1570 (61.9%)	657 (60.6%)	
T2	1393 (38.5%)	966 (38.1%)	427 (39.4%)	
Surgery				0.213
Yes	2537 (70.1%)	1793 (70.7%)	744 (68.6%)	
No	1083 (29.9%)	743 (29.3%)	340 (31.4%)	
Radiotherapy				0.823
Yes	51 (1.4%)	35 (1.4%)	16 (1.5%)	
No	3569 (98.6%)	2501 (98.6%)	1068 (98.5%)	
Chemotherapy				0.340
Yes	1148 (31.7%)	792 (31.2%)	356 (32.8%)	
No/unknown	2472 (68.3%)	1744 (68.8%)	728 (67.2%)	
Tumor size				0.567
<3 cm	1777 (49.1%)	1237 (48.8%)	540 (49.8%)	
3–5 cm	1843 (50.9%)	1299 (51.2%)	544 (50.2%)	
Insurance				0.786
Any medicaid	773 (21.4%)	547 (21.6%)	226 (20.8%)	
Insured	2761 (76.3%)	1931 (76.1%)	830 (76.6%)	
Uninsured	86 (2.4%)	58 (2.3%)	28 (2.6%)	
Marital status				0.175
Married	2118 (58.5%)	1499 (59.1%)	619 (57.1%)	
Single	701 (19.4%)	501 (19.8%)	200 (18.5%)	
Divorced/separated	518 (14.3%)	347 (13.7%)	171 (15.8%)	
Widowed	283 (7.8%)	189 (7.5%)	94 (8.7%)	
County‐level median household income^b^				0.098
77.04–110.97 K	912 (25.2%)	652 (25.7%)	260 (24.0%)	
61.03–77.03 K	832 (23.0%)	554 (21.8%)	278 (25.6%)	
54.36–61.02 K	989 (27.3%)	701 (27.6%)	288 (26.6%)	
19.26–54.35 K	887 (24.5%)	629 (24.8%)	258 (23.8%)	

Abbreviation: HCC, hepatocellular carcinoma.

^a^ Chi‐square test.

^b^ Shown in U.S. dollars.

### 3.2 Univariate and multivariate analyses

**FIGURE 2:**
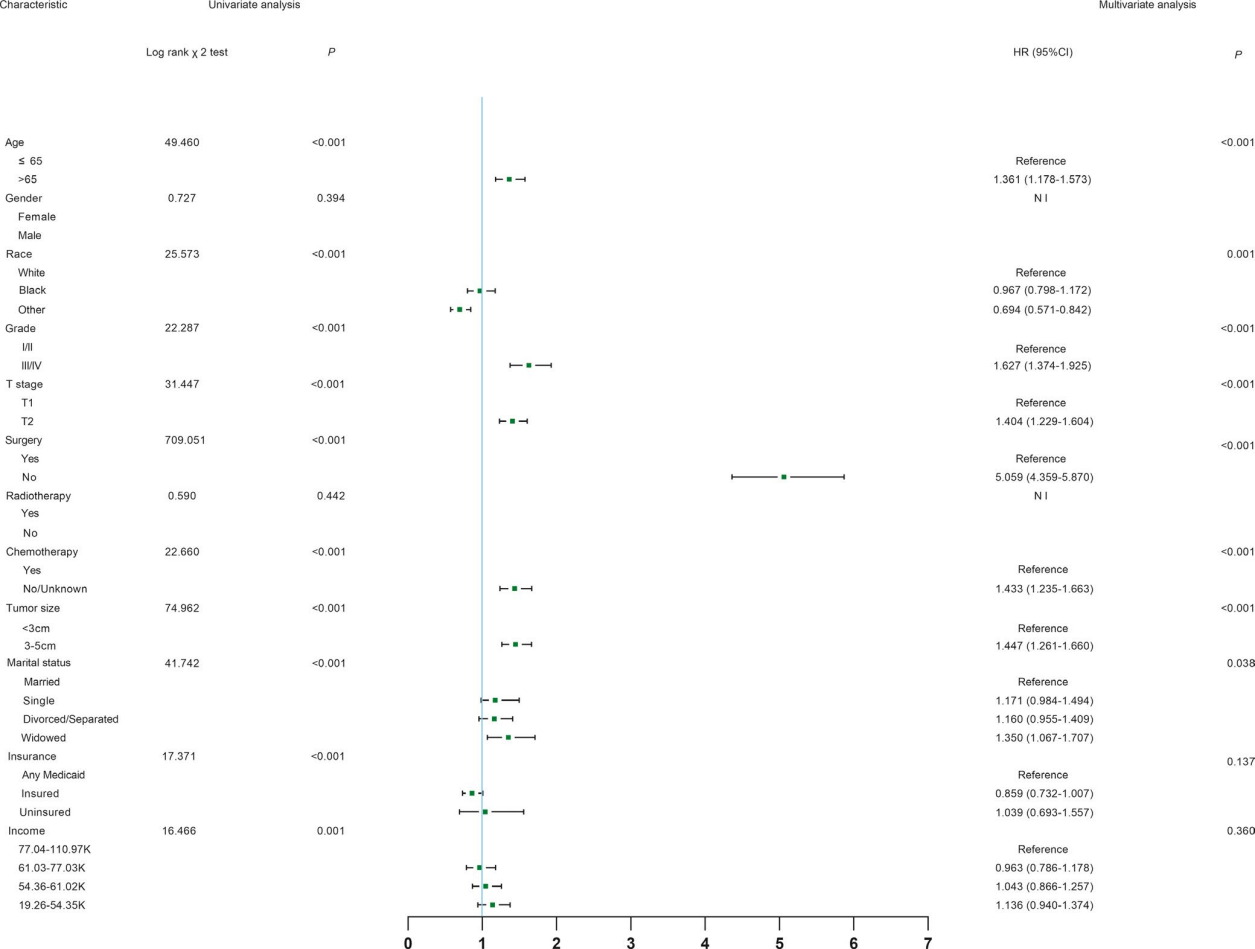
Univariate analysis and forest plot of the hazard ratio of hepatocellular carcinoma cancer‐specific survival based on the training cohort. HR, hazard ratio; CI, confidence interval

Age, race, grade, T stage, surgery, chemotherapy, tumor size, marital status, insurance, and income were significantly (*p* < 0.05) identified in univariate analysis in the training cohort (Figure 2). The multivariable analysis showed that age (*p* < 0.001), race (*p* = 0.001), grade (*p* < 0.001), T stage (*p* < 0.001), surgery (*p* < 0.001), chemotherapy (*p* < 0.001), tumor size (*p* < 0.001), and marital status (*p* = 0.038) were independent prognostic factors for CSS (Figure 2), which were included in the nomogram.

### 3.3 Construction and validation of nomogram

**FIGURE 3:**
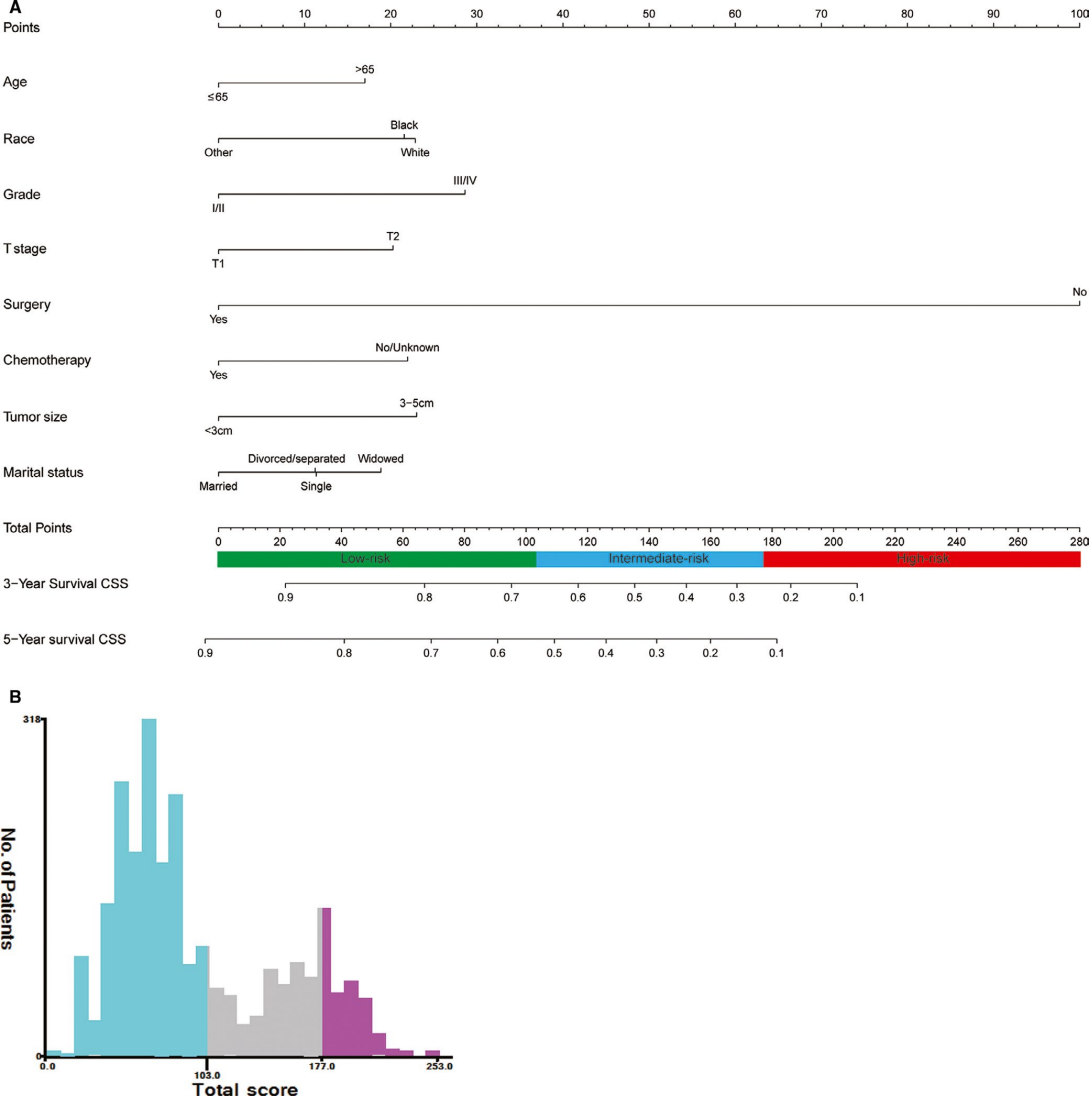
CSS‐associated nomogram for early hepatocellular carcinoma patients and risk stratification based on nomogram. (A) Nomogram for 3‐, 5‐year CSS; (B) Range of risk stratification based on total score. CSS, cancer‐specific survival

**FIGURE 4:**
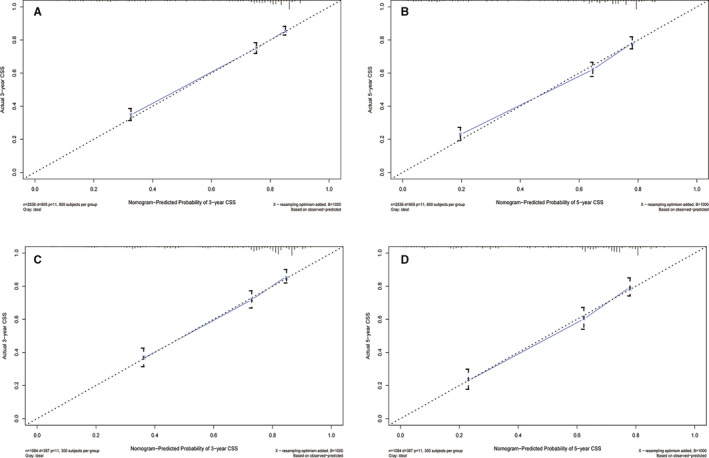
Calibration plots of CSS‐associated nomogram. (A) Calibration plot of 3‐year CSS in the training cohort; (B) Calibration plot of 5‐year CSS in the training cohort; (C) Calibration plot of 3‐year CSS in the validation cohort; (D) Calibration plot of 5‐year CSS in the validation cohort. CSS, cancer‐specific survival

**FIGURE 5:**
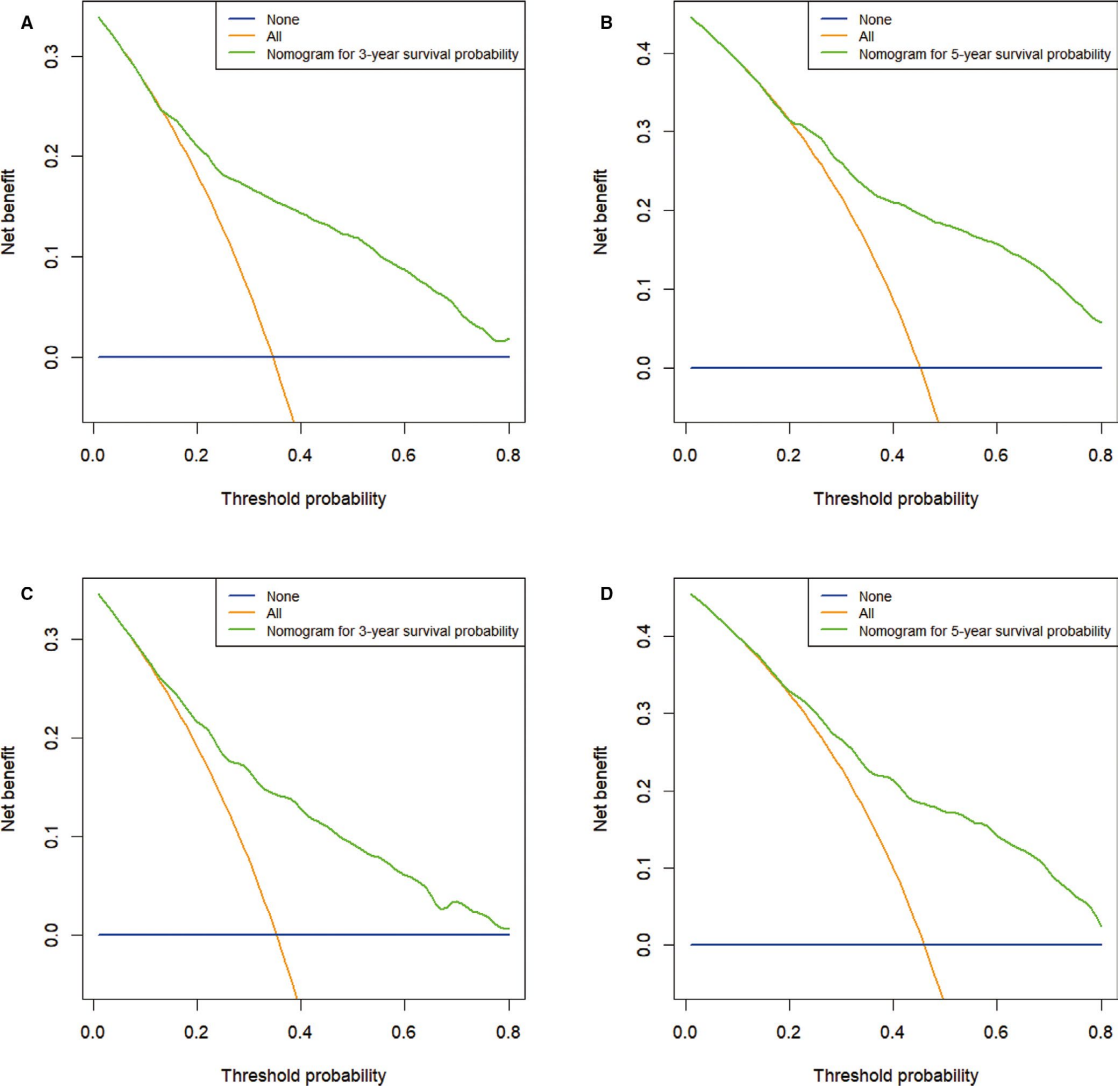
Decision curve analysis of CSS‐associated nomogram. (A) DCA curve of 3‐year CSS in the training cohort; (B) DCA curve of 5‐year CSS in the training cohort; (C) DCA curve of 3‐year CSS in the validation cohort; (D) DCA curve of 5‐year CSS in the validation cohort. The blue line represents that no patients had cancer‐specific deaths, and the orange line represents that cancer‐specific death occurred in all patients. The green line represents the net benefit of using the nomogram in the prediction of survival. The x‐axis represents the threshold probabilities, and the y‐axis represents the net benefit. DCA, decision curve analysis; CSS, cancer‐specific survival

A nomogram was established based on these significant prognostic factors for CSS. The nomogram was virtually displayed for predicting the 3‐, 5‐year CSS (Figure 3A), and was validated internally. The nomogram‐related C‐indexes in the training and validation cohorts were 0.755 (95% CI: 0.739–0.771), 0.737 (95% CI: 0.712–0.762), respectively. However, for the TNM stage, the C‐indexes of were 0.552 (95% CI: 0.534–0.570) and 0.567 (95% CI: 0.542–0.592) in the training and validation cohorts, respectively (Table S1). The calibration curves manifested good consistency in the probability of 3‐, 5‐years CSS between the actual observation and the nomogram prediction in the training (Figure 4A and B; respectively) and validation (Figure 4C and D; respectively) cohort. Similarly, the 3‐, 5‐year AUCs in the training cohort were 0.783 and 0.779, respectively (Figure S1A and B), the 3‐, 5‐year AUCs in the validation cohort were 0.767 and 0.776, respectively (Figure S1C and D). Furthermore, the nomogram‐related DCA curves of 3‐, 5‐ year CSS in the training (Figure 5A and B) and validation (Figure 5C and D) cohorts also showed good clinical application potential, revealing preferable positive net benefit. Meanwhile, compared with the AJCC TNM staging system, the CSS nomogram had better clinical practicality (Figure [Link], [Link]).

### 3.4 Risk stratification system according to the nomogram model

**FIGURE 6:**
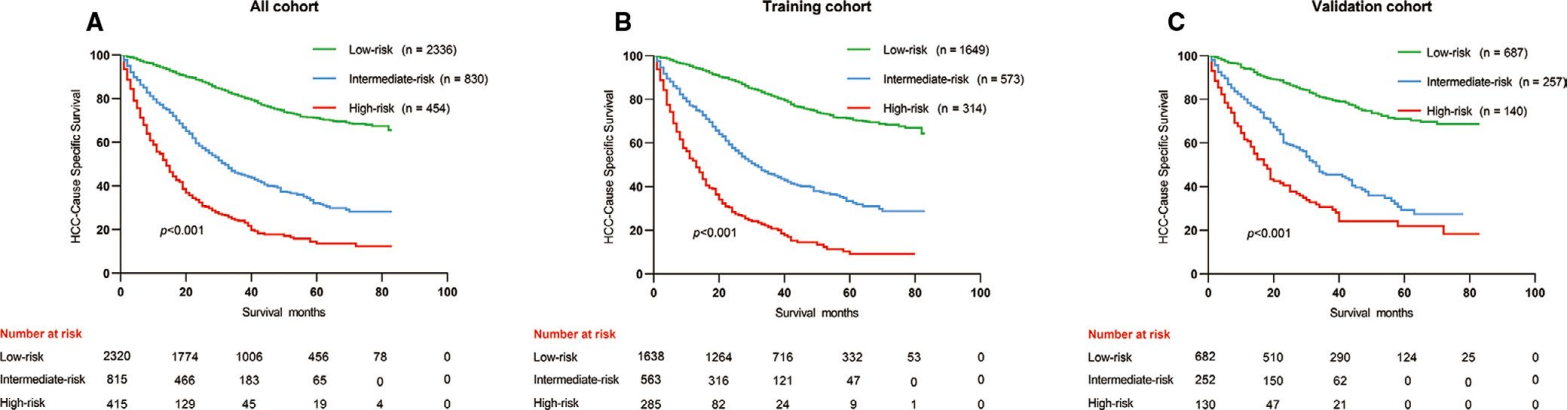
Kaplan–Meier curves of CSS for risk classification based on the nomogram scores. (A) In all cohort; (B) In the training cohort; (C) In the validation cohort. CSS, cancer‐specific survival

Along with the nomogram was generated, we also developed a risk stratification system based on cutoff value of the total scores of each patient in the training cohort (Figure 3B). All patients were grouped into the low‐risk (score: 0–103), intermediate‐risk (score: 104–177), and high‐risk groups (score: 178–253). In all cohort, Kaplan–Meier analysis revealed that the 3‐years CSS rates were 81.2%, 45.6%, and 24.0%, respectively, in low‐, intermediate‐, and high‐risk groups, and the 5‐years CSS rates were 71.3%, 32.1%, and 14.4%, respectively, in low‐, intermediate‐, and high‐risk groups (both *p* < 0.001, Figure 6A). In the training cohort, the 3‐years CSS rates were 81.5%, 45.7%, and 20.8%, respectively, in low‐, intermediate‐, and high‐risk groups, and the 5‐years CSS rates were 71.2%, 33.4%, and 9.2%, respectively, in low‐, intermediate‐, and high‐risk groups (both *p* < 0.001, Figure 6B). In the validation cohort, the 3‐years CSS rates were 80.4%, 45.4%, and 30.7%, respectively, in low‐, intermediate‐, and high‐risk groups, and the 5‐years CSS rates were 71.0%, 29.3%, and 21.9%, respectively, in low‐, intermediate‐, and high‐risk groups (both *p* < 0.001, Figure 6C).

## 4 DISCUSSION

In our study, through the analysis of patients’ baseline demographic and clinical characteristics, we developed and validated the prognostic nomogram models for 3‐, 5‐ years CSS of patients with early HCC, which could make for the clinical prognostic evaluation, management of high‐risk patients, and personalized treatment.

In our study, through univariate and multivariate analyses, we found numerous factors significantly affected CSS, including age, race, marital status, grade, T stage, tumor size, surgery, and chemotherapy. Although, we recognized that several variables, including gender, radiotherapy, insurance, and income were not identified as prognostic significance. In early HCC patients, potential prognostic significant factors different from those in normal HCC patients, it also suggests that we should further explore for the differences in potential prognostic factors between early and normal HCC.

There is some controversy about surgical treatment and other treatments for patients with early HCC.^13^, ^20^, ^21^, ^22^ A propensity‐matched analysis showed that compared with stereotactic body radiotherapy, surgical resection provided better survival advantage in patients with small HCC (tumor size ≤3 cm).^20^ Mills et al. found that surgical resection provided better survival compare with thermal ablation in patients with localized HCC.^21^ Moreover, a 10‐Year SEER‐Medicare Analysis study demonstrated that using surgical therapy to facilitate the survival of patients with early HCC.^13^ However, Yamakado et al. found that patients with early‐stage HCC (tumor size ≤5 cm) by hepatectomy provided overall survival (OS) and disease‐free survival (DFS) had no significant difference, compared with those achieved by radiofrequency (RF) ablation combined with chemoembolization.^22^ Based on the result of our research, among all variables included in the nomogram, surgery was the most remarkable prognosticator for CSS. This result indicated the importance of surgical treatment in early stage HCC. Therefore, surgical treatment should be chosen as a highly effective option for patients with early HCC if conditions permit. In addition, chemotherapy was also found to improve survival rates for early HCC in our study. An analysis of the START trial showed that the incorporation of transcatheter arterial chemoembolization (TACE) with sorafenib could promote the patients with early–intermediate stage HCC for survival rate,^23^ which confirmed our findings.

Among tumor features (pathological grade, T stage, and tumor size) variables included in the nomogram, the influence of pathological grade for CSS is the largest. Margonis GA and colleagues indicated that poorly differentiated HCC was associated with worse prognosis.^24^ A survival study of HCC patients undergoing surgery indicated the grade was an independent predictor in patients with HCC for the 5‐year survival rate.^11^ Moreover, a large randomized controlled trial (RCT) showed that tumor differentiation could influence OS and cancer‐free survival in HCC patients with performed percutaneous radiofrequency thermal ablation.^25^ Consistently, in our study, patients with good differentiation have a better prognosis. Tumor size is also a critical feature of carcinoma, and it may affect cancer survival. A recent study showed that tumor size ≤3 cm is low malignant potential.^14^ Meanwhile, a prospective study by Camma et al. indicated that HCC patients with tumors smaller than 3 cm had better survival after treatment.^26^ Similarly, our study suggested that tumor size less than 3 cm has a survival advantage over that of 3–5 cm. As is known to all, the prognosis of most solid tumors was predicted according to the AJCC‐TNM stage,^27^ which is also reflected in our results.

In addition, baseline demographics variables (age, race, and marital status) were identified as prognosticators of HCC. Some studies showed young patients with early HCC had a better prognosis,^8^, ^28^ which is similar to our results. A previous study on predictive of early HCC based on SEER found that whites have better survival rates than blacks, and other (American Indian/Alaskan Native, Asian/Pacific Islander) races have better survival than blacks and whites.^29^ However, our results showed other (American Indian/Alaskan Native, Asian/Pacific Islander) races had the highest survival and blacks had better survival rates than whites in this study. The different outcomes may be mainly due to their different inclusion criteria. The previous study focused on patients who had received liver resection, while our study payed attention to all patients who had received or not received liver resection. Married patients with HCC often have an advantage for survival.^10^, ^18^ Similar findings were made in our study, married patients had the best survival, widows the worst survival.

Up to now, there are several staging systems have been constructed about HCC, such as Barcelona Clinic Liver Cancer (BCLC) staging, American Joint Committee on Cancer (AJCC) TNM staging, Chinese University Prognostic Index (CUPI), and so on. The above staging systems are generally more practical for the prognosis of all HCC stages. However, our nomogram shows a satisfactory ability of predicting the CSS of early stage HCC. Santambrogio et al. revealed that C‐indexes of BCLC staging system for early HCC in the training and validation cohort were 0.6479 and 0.6323, respectively, and AUCs in the training and validation cohort were 0.5949 and 0.5873, respectively.^30^ A study about 379 patients with early HCC found C‐indexes were 0.51, 0.59, in BCLC and AJCC staging systems, respectively.^31^ As is known to all, when the model‐related C‐index and AUC overtop 0.7, it indicates that the prediction model has relatively good resolving ability.^32^ In our study, the nomogram‐related C‐index was 0.755, higher than the AJCC TNM stage (0.552), which presented a better predictive performance. There are also some nomogram models for predicting the survival prognosis of early stage HCC,^17^, ^29^ above studies focused on patients with early HCC for overall survival (OS) and disease‐free survival (DFS). However, prognostic nomogram for patients with early stage HCC for CSS may not have been adequately developed and validated.

Based on the nomogram, the formation of a risk stratification system, which could distinctly divide all patients into three risk prognostic groups. In addition, we also drew DCA curves to assess the clinical practicability of nomogram. A study indicated that the DCA curve could evaluate whether medical decisions and strategies based on nomogram could facilitate survival prognosis of patients to reflect the clinical value of the nomogram.^33^ Of note, there are several restrictions. First, our study was performed by retrospective analysis. Second, the nomogram was constructed based on the SEER database. It is not necessarily representative of the rest of the world. Third, there is lack of some important information in the SEER database, such as the cause of HCC, some blood and inflammatory indicators, which could cause results bias. Finally, although the nomogram and risk classification system had commendably reflected predictive performance in two subgroups, externally validation for the predictive model is still required.

In summary, based on the significant risk factors identified in the analysis, we developed a practical and reliable nomogram for predicting the CSS of the early HCC patients. This nomogram was helpful to guide clinicians to individualize the treatment for patients with early HCC, and provided the basis for the clinical management of high‐risk patients, showing great potential for clinical application. Despite internal validation was performed, external validation on the relevant early HCC data set should be considered.

## CONFLICT OF INTEREST

The authors have no conflicts of interest to declare.

## ETHICAL APPROVAL

The approval process of Institutional Review Board was waived because of the de‐identified information of the patients included in the Surveillance, Epidemiology, and End Results database.

## Supporting information

Fig S1

Fig S2

Table S1

## Data Availability

The data that support the findings of this study are available from the corresponding author upon reasonable request.
