# Supplementary material for: A practical nomogram and risk stratification system predicting the cancer‐specific survival for patients with early hepatocellular carcinoma
Source: Cancer Med. 2020 Dec 6;10(2):496–506. doi: 10.1002/cam4.3613 (PMC7877377; doi:10.1002/cam4.3613)

Figure S1. ROCs of CSS associated nomogram. (A) ROC of 3-year CSS in the training cohort; (B) ROC of 5-year CSS in the training cohort; (C) ROC of 3-year CSS in the validation cohort; (D) ROC of 5-year CSS in the validation cohort. ROC, receiver operating characteristics curve; CSS, cancer-specific survival.


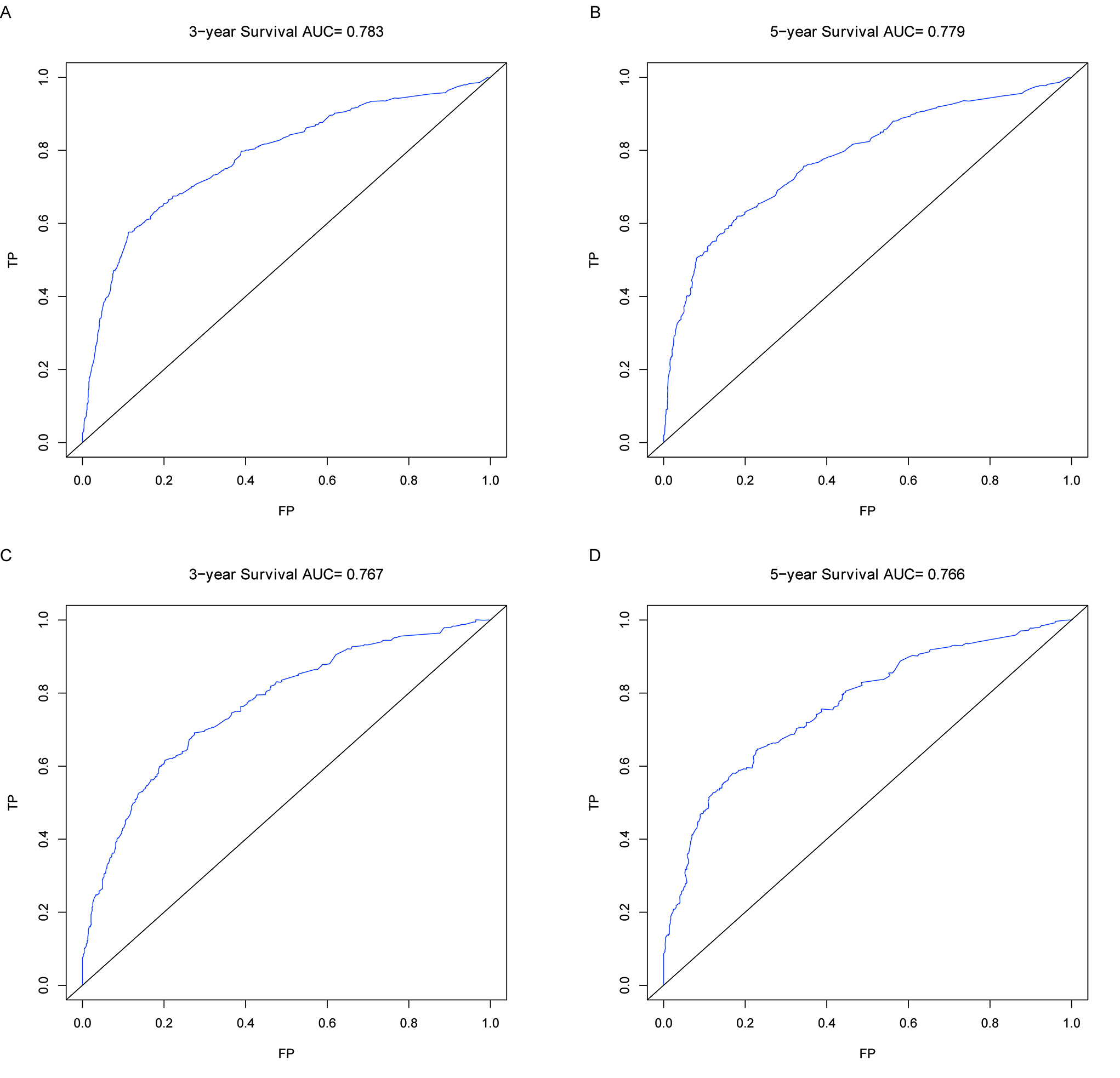

Supplement: Supplementary file 1 — Fig S1 [file CAM4-10-496-s001.docx]
