# Supplementary material for: A practical nomogram and risk stratification system predicting the cancer‐specific survival for patients with early hepatocellular carcinoma
Source: Cancer Med. 2020 Dec 6;10(2):496–506. doi: 10.1002/cam4.3613 (PMC7877377; doi:10.1002/cam4.3613)

Figure S2. Decision curve analysis of comparison between nomogram and TNM stage for predicting survival. (A) Comparison in the training cohort; (B) Comparison in the validation cohort. The blue line indicates the net benefit using the TNM staging system. The green line represents the net benefit using the nomogram.


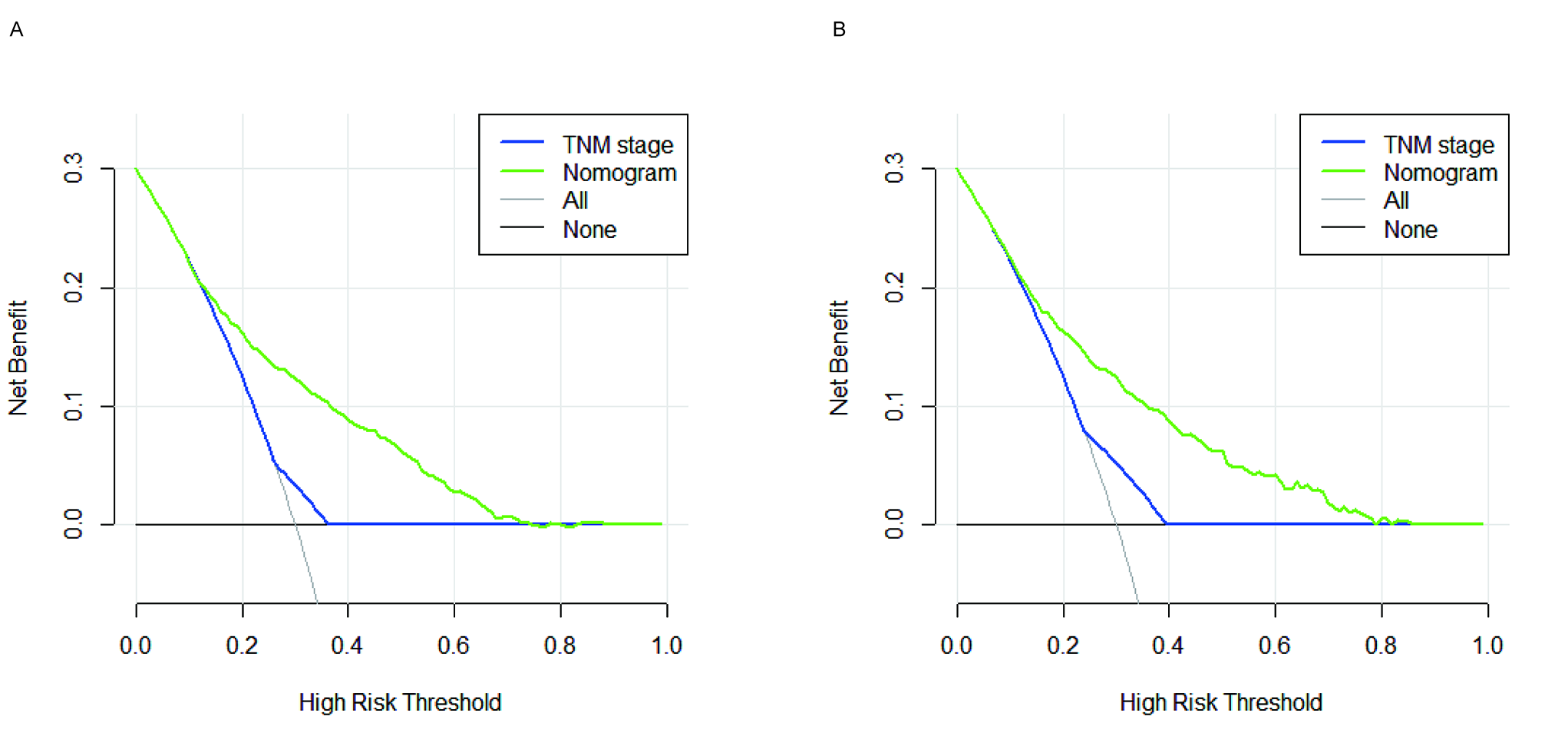

Supplement: Supplementary file 2 — Fig S2 [file CAM4-10-496-s002.docx]
