# Supplementary material for: A practical nomogram and risk stratification system predicting the cancer‐specific survival for patients with early hepatocellular carcinoma
Source: Cancer Med. 2020 Dec 6;10(2):496–506. doi: 10.1002/cam4.3613 (PMC7877377; doi:10.1002/cam4.3613)
Supplement: Supplementary file 3 — Table S1 [file CAM4-10-496-s003.docx]

**Table S1.** Comparison of C-indexes between the Nomogram and TNM stage in patients with early HCC

| Survival | Tumor stage types | Training | cohort | Validation | cohort |
| --- | --- | --- | --- | --- | --- |
|  |  | C-index | HR (95% CI) | C-index | HR (95% CI) |
| CSS | Nomogram | 0.755 | 0.739-0.771 | 0.737 | 0.712-0.762 |
| CSS | TNM stage | 0.552 | 0.534-0.570 | 0.567 | 0.542-0.592 |

Abbreviations: TNM, tumor-node-metastasis; HCC, hepatocellular carcinoma; CSS, cancer-specific survival; HR, hazard ratio; CI, confidence intervals.
